# CE‐MS for metabolomics: Developments and applications in the period 2018–2020

**DOI:** 10.1002/elps.202000203

**Published:** 2020-10-04

**Authors:** Wei Zhang, Rawi Ramautar

**Affiliations:** ^1^ Biomedical Microscale Analytics, Leiden Academic Centre for Drug Research Leiden University Leiden The Netherlands

**Keywords:** Biomedical and clinical, Mass spectrometry, Metabolomics, microbial and plant, Technological developments

## Abstract

Capillary electrophoresis‐mass spectrometry (CE‐MS) is now a mature analytical technique in metabolomics, notably for the efficient profiling of polar and charged metabolites. Over the past few years, (further) progress has been made in the design of improved interfacing techniques for coupling CE to MS; also, in the development of CE‐MS approaches for profiling metabolites in volume‐restricted samples, and in strategies that further enhance the metabolic coverage. In this article, which is a follow‐up of a previous review article covering the years 2016–2018 (*Electrophoresis* 2019, 40, 165–179), the main (technological) developments in CE‐MS methods and strategies for metabolomics are discussed covering the literature from July 2018 to June 2020. Representative examples highlight the utility of CE‐MS in the fields of biomedical, clinical, microbial, plant and food metabolomics. A complete overview of recent CE‐MS‐based metabolomics studies is given in a table, which provides information on sample type and pretreatment, capillary coatings, and MS detection mode. Finally, some general conclusions and perspectives are given.

**Abbreviations**
AA: amino acid
ACN: acetonitrile
AD: Alzheimer's disease
CESI: capillary electrophoresis with sheathless interface
CS: chiral selector
DCIS: ductal carcinoma *in situ*
FASI: field amplified sample injection
GF: germ‐free
GSH: reduced glutathione
HMT: Human Metabolome Technologies
IBD: inflammatory bowel disease
ID: internal diameter
IDC: invasive ductal carcinoma
LDIS: large volume dual‐preconcentration by isotachophoresis and stacking
MeOH: methanol
MSI: multisegment injection
NACE: nonaqueous capillary electrophoresis
NM: nanomaterial
OD: outer diameter
PCA: principal component analysis
SPF: specific pathogen‐free
tITP: transient isotachophoresis

## 1 Introduction

In metabolomics, CE‐MS is a strong analytical separation technique for the efficient profiling of polar and charged metabolites, especially for compound classes, such as amino acids (AA), nucleotides, small organic acids, and sugar phosphates [1, 2, 3, 4]. The use of this approach, however, for metabolic profiling studies is underrepresented in comparison to other analytical separation techniques [5, 6]. The metabolomics and separation science community still consider CE‐MS as technically challenging and less reproducible in comparison to GC‐MS and LC‐MS [7]. The limited use of CE‐MS may also be attributed to the lack of standard operating procedures and workflows despite new developments in sample throughput and quality control [8, 9].

Over the past few years, various research groups have clearly demonstrated the value of CE‐MS for biomarker discovery studies using large (clinical) sample sets. For example, Mischak and co‐workers have profiled native peptides in more than 20000 human urine samples by CE‐MS at different laboratories with an acceptable interlaboratory reproducibility [10, 11, 12, 13]. Harada et al. evaluated the analytical performance of CE‐MS for metabolic profiling of more than 8000 human plasma samples from the Tsuruoka Metabolomics Cohort Study over a 52‐month period [14]. The study provided an absolute quantification for 94 polar and charged metabolites in plasma with a reproducibility comparable to other analytical platforms. CE‐MS has also shown good mutual agreement (mean bias < 15%) for reliable quantification of various plasma or serum metabolites and fatty acids (FAs) as compared to RP LC‐MS and GC‐MS [15, 16]. Overall, these studies clearly demonstrate the (added) value of CE‐MS in metabolomics.

In this article, which is a follow‐up of our previous CE‐MS‐based metabolomics reviews [17, 18, 19, 20, 21, 22], an overview of recent developments in CE‐MS approaches for metabolomics is provided as reported over the past 2 years. Attention will be paid to main technological developments including new interfacing designs and CE‐MS approaches for volume‐restricted metabolomics. Also strategies for further improving the metabolic coverage of CE‐MS will be outlined. The recent CE‐MS‐based metabolomics studies are summarized in a Table and selected representative examples will be highlighted in order to show the usefulness of CE‐MS in the fields of biomedical, clinical, microbial, plant and food metabolomics. Finally, some general conclusions and perspectives are provided.

## 2 Technological Developments

**Figure 1:**
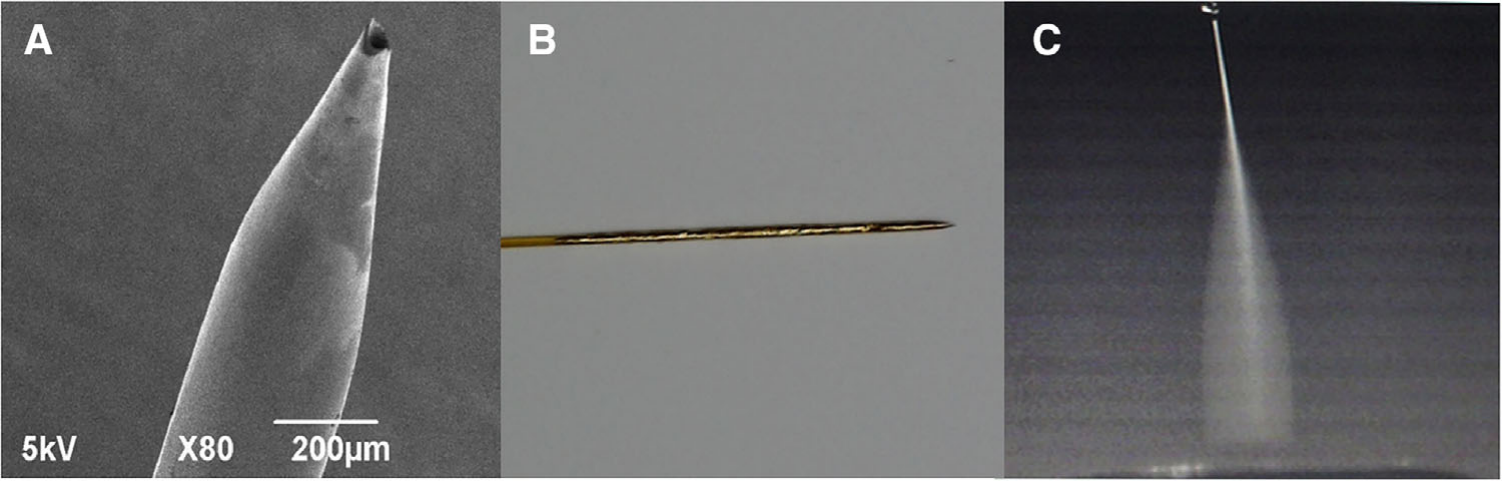
(A) The surface morphology of the gold foil covered emitter used for sheathless CE‐MS as observed by SEM and (B) photo of the emitter tip, and (C) the electrospray plume produced by the emitter under application of an electrical field. Reproduced from Ref. [23] with permission.

Coupling CE to MS is an active research field, aiming to help obtain stable, reproducible, and sensitive analysis with interfaces compatible with CE instrumentation. Recently, Zhang et al. reported the design and performance of a sheathless CE‐MS interface [23]. This design utilized a 70‐cm long fused‐silica capillary with 3 mm long bare fused silica at one end exposed by burning and subsequently etched in hydrofluoric acid to form a symmetrical tapered tip. The tip was then washed and dried before being evenly smeared with quick‐drying epoxy glue and quickly twined with a piece of gold foil (Fig. 1A and B). The sheathless interface is ready to use once the epoxy glue dries. The constructed emitter is smooth and flat, thus, easy to form a stable electrospray plume (Fig. 1C). The authors flushed the capillary alternatively with hexadimethrine bromide (HDB) and dextran sulfate (DS) solutions to form three‐layered coatings for the analysis of organic acids, and four‐layered coatings for the analysis of cationic analytes. The evaluation of the interface performance was investigated and a stable MS signal was obtained when the flowrate was in the range of 80 to 510 nL/min and the ESI voltage in range of 1.9 to 2.2 kV. The applicability was demonstrated with the analysis of four alkaloids, using 20 mM ammonium formate in 50% v/v acetonitrile (ACN) (pH 3.0). For the tested compounds, absolute LOD values below 1 fmol could be acquired with satisfactory migration‐time repeatability. Additionally, this work also included the application of this setup for the analysis of organic anions. The ease in manufacturing of this proposed interface and its capability to separate cations and anions render it a very promising tool for metabolomics studies.

**Figure 2:**
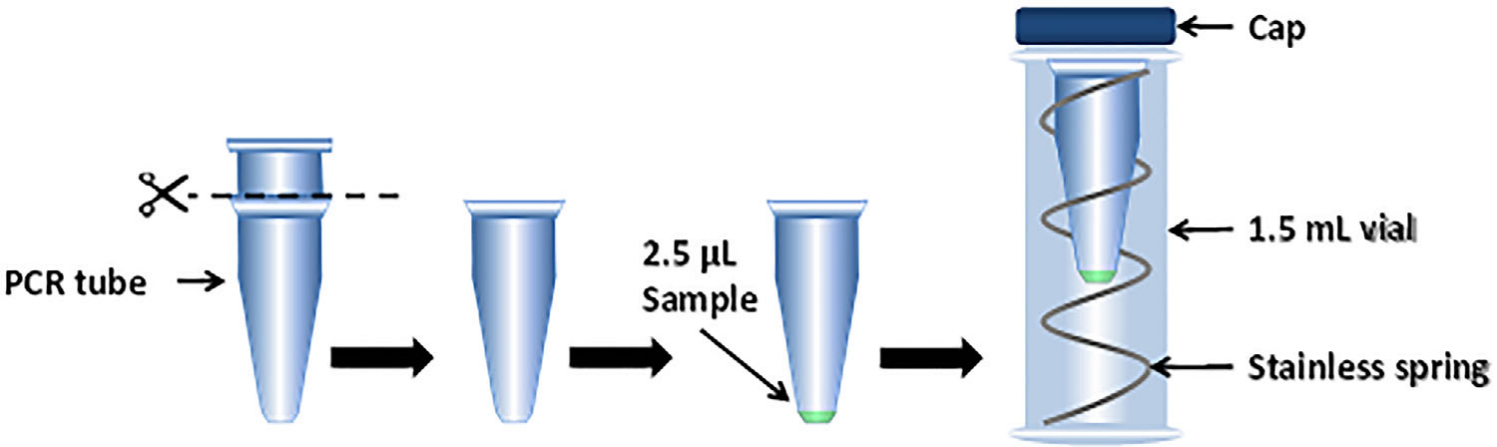
Schematic overview of a modified CE vial used for metabolic profiling of low‐volume samples by CE‐MS. The top of a PCR microtube was removed and placed in a regular Sciex CE sample vial with the support of stainless springs. Reproduced from Ref. [27] with permission.

A major challenge in metabolomics is still the efficient analysis of polar and charged metabolites in biological samples, even more so when such samples are limited in volume or biomass. Over the past few years, CE‐MS emerged as a strong analytical technique for the profiling of metabolites in small‐volume biological samples, ranging from body fluids of small animal models to low numbers of mammalian cells [24, 25, 26]. However, the typical volume needed in sample vials of (commercial) CE instruments is in the order of 5 to 10 (or more) microliters, while only nanoliters are injected into the CE system. Therefore, this volume mismatch may limit the analytical performance of CE‐MS for volume‐restricted metabolomics studies. To mitigate this dilemma, Sánchez‐López et al. modified CE vials in‐house by removing the top of PCR microtubes and placing them in regular Sciex sample vials with the support of stainless springs [27]. This modification, shown in Fig. 2, helped reduce the necessary final sample volume to as little as 2.5 µL per sample. The utility of such a design was exemplified with the analysis of biomass‐limited tissue material, namely, 20 µm‐thick mouse kidney tissue sections, aimed at discovering metabolic changes related to polycystic kidney disease (PKD). During the analysis, the authors also adopted the use of dopant enriched nitrogen (DEN)‐gas, which was delivered as a coaxial sheath flow around the ESI emitter via an in‐house fabricated polymer cone, to further improve the detection sensitivity of sheathless CE‐MS using hydrodynamic sample injection. This study led to the detection of 112 cationic metabolite features that had relative standard deviation (RSD) for peak areas below 30% across the QC samples and different experimental groups could be clearly separated based on the obtained metabolic profiles.

**Figure 3:**
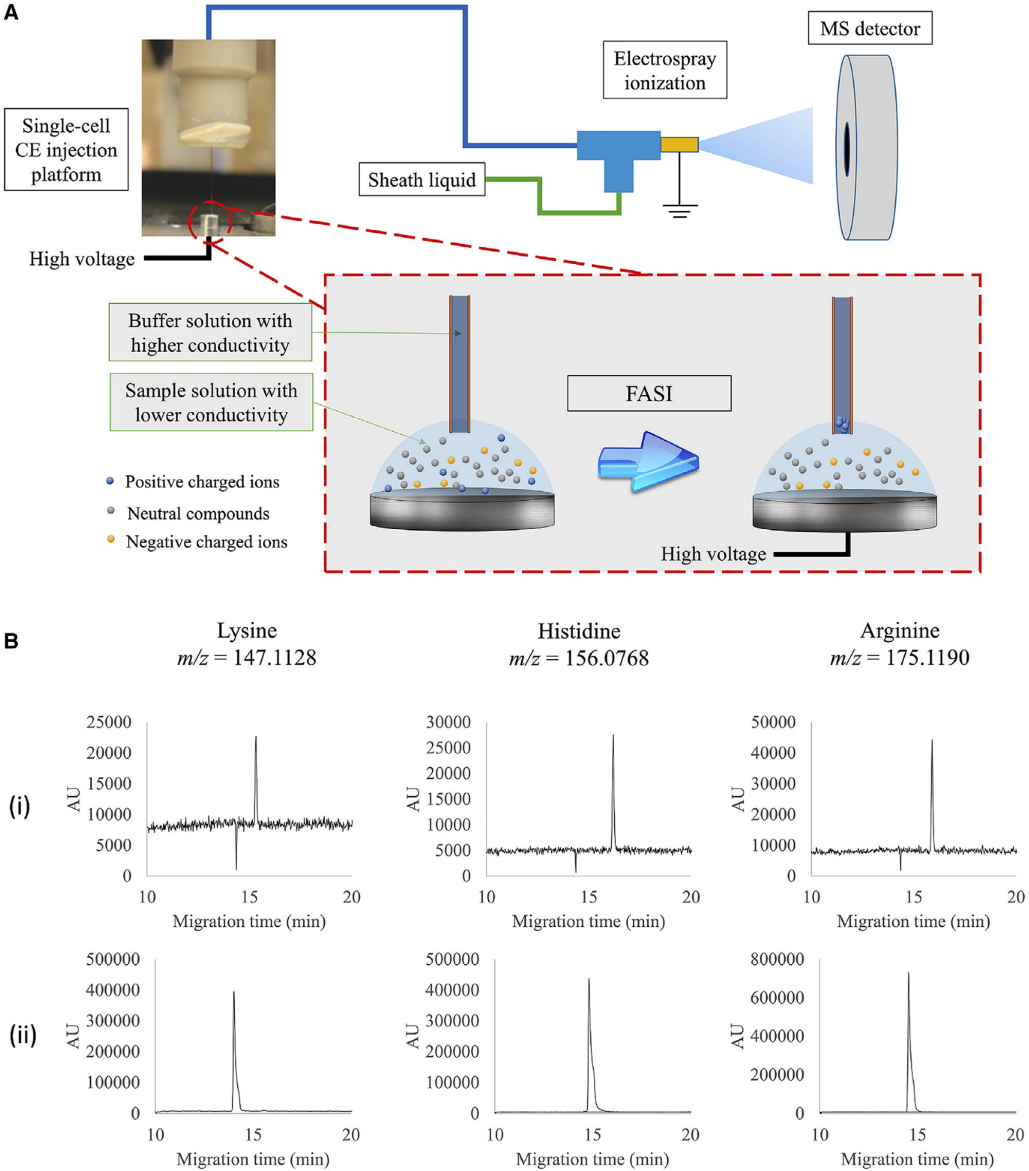
Comparison of hydrodynamic injection and FASI for CE‐MS analysis of a lysine, histidine, and arginine mixture. (A) System schematic. (B) The extracted ion electropherograms of lysine, histidine, and arginine standards obtained with CE‐MS using (i) 6 nL hydrodynamic injection of 100 nM lysine, histidine, and arginine solution and (ii) FASI of 10 nM lysine, histidine, and arginine standard solution. Reproduced from Ref. [28] with permission.

Minimizing the final sample volume is one way to tackle the volume mismatch, however, a more elegant approach would be to consider to online preconcentration/stacking procedures, especially for the emerging field of single‐cell metabolomics. In order to accurately capture intracellular metabolites of diverse concentrations, Liao et al. reported a CE‐MS method that combines field amplified sample injection (FASI), sample desalting, and low‐volume manipulations to measure metabolites in an extract from a single cell [28]. In this work, the analysis was conducted on an in‐house assembled CE coupled to a high‐resolution MS system via a custom‐built co‐axial sheath‐liquid ESI source, using 1% formic acid (FA) as BGE. FASI was conducted by electrokinetic injection of the sample solution (which had a total volume 500 nL) at 20 kV for 30 s and a schematic overview is given in Fig. 3A. To balance the stacking efficiency and the extent of Joule heating, the authors optimized the sample matrix composition and discovered the most effective to be a mixture of formic acid:water:methanol (0.01/4.99/95, v/v/v). FASI with such a sample matrix provided 307‐, 191‐, and 215‐fold detection sensitivity enhancement, separately, for analyzing standard mixtures of lysine, histidine, and arginine, when compared to hydrodynamic injection, as illustrated in Fig. 3B. Moreover, a salt precipitation step was introduced for cell lysates, making use of the insolubility of salts in a mixture of isopropanol: ACN (4:1), which led to an improved signal intensity by 4.5‐ to 6‐fold for endogenous lysine, histidine, and arginine. The impact of migration time shift on metabolite identification was minimized in this work by employing a two internal‐standard normalization. This method was then examined using a pool of 21 cations, and acceptable results were obtained for aspects of linearity and repeatability. The obtained LOQ values (*S*/*N* = 5) ranged between 0.2 and 3.6 nM. The capability of the proposed FASI CE‐MS system was demonstrated by the analysis of single neurons (∼50 µM diameter) isolated from the *Aplysia* nervous system, where 37 cationic metabolites could be detected and identified.

**Figure 4:**
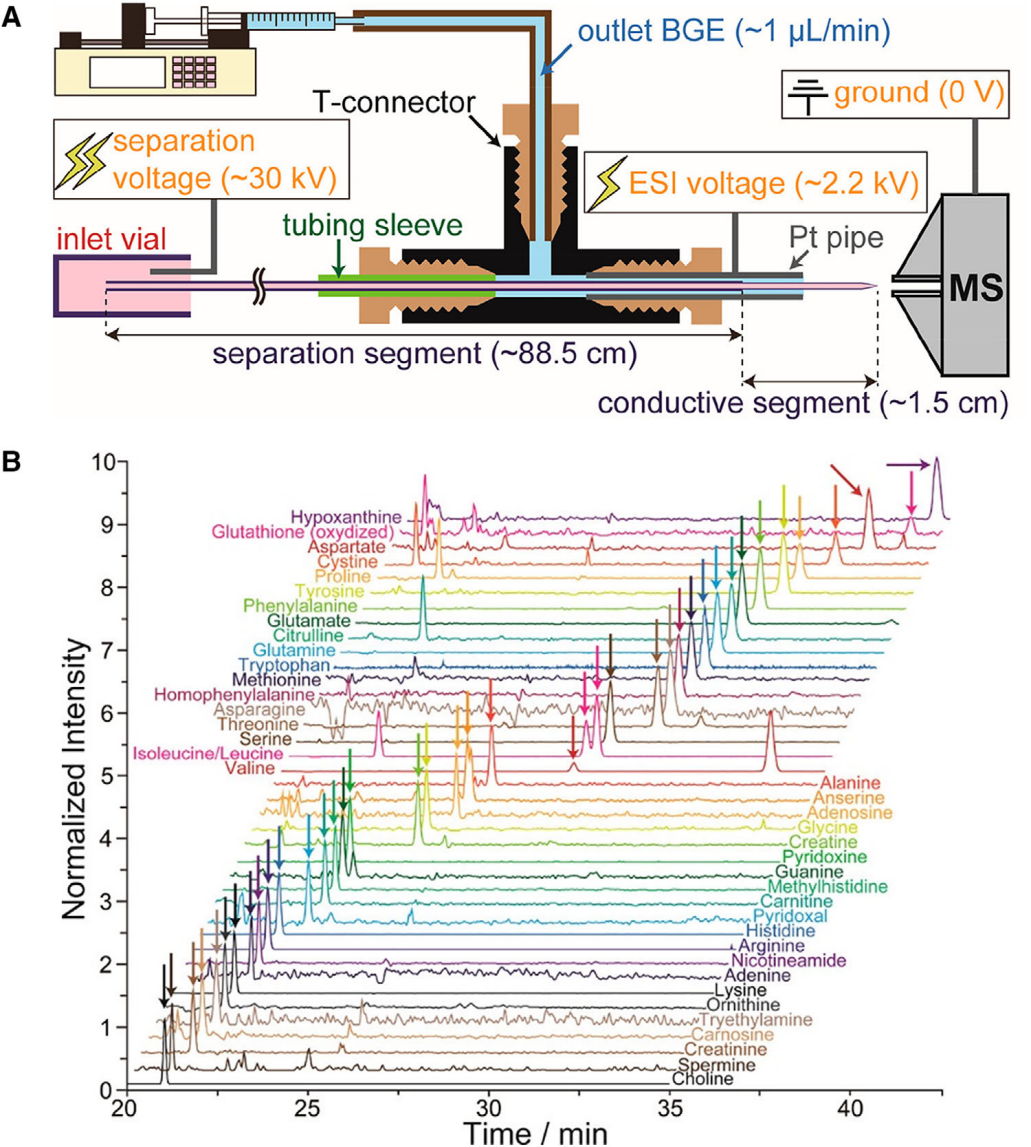
(A) Schematic overview of the nano CESI‐MS setup including experimental conditions. (B) Extracted ion electropherograms obtained for the analysis of 40 metabolites in an extract of a single HeLa cell by LDIS in combination with CE‐TOF‐MS. Reproduced from Ref. [29] with permission.

Another aspect that can contribute to single cell metabolomics studies is by optimizing CE‐MS interfacing techniques to achieve more efficient ionization, which in combination with preconcentration techniques will greatly improve the capability of CE‐MS in handling single cells. To enable adequate sensitivity in metabolic profiling of single human cells, Kawai et al. [29] incorporated a “nanocapillary electrophoresis with sheathless interface (CESI)” emitter with a large‐volume dual preconcentration technique. The fabrication of a “nanoCESI” emitter utilized fused‐silica capillary (internal diameter/outer diameter [ID/OD], 50 µm/360 µm), with the polyimide at one end etched away in hydrofluoric acid (HF) till the wall thickness reduced to 20–30 µm. The etched segment was then tapered by a CO_2_ laser puller before it was subjected to further hydrofluoric acid (HF) etching until the wall thickness became 10 µm and the average bore size 9.3 µm. Figure 4A depicts the experimental condition used in this work, consisting of low‐pH separation conditions and MS detection in positive‐ion mode. The employed setup offered a sensitivity improvement of between 1.1‐ and 3.5‐fold for 20 AA tested when compared to conventional CESI. The authors then employed an online sample enrichment method, that is, large volume dual‐preconcentration by isotachophoresis and stacking (LDIS), which allows an injection volume of ca. 1200 nL (out of 5 µL sample volume), which resulted in LOD values between 0.45 and 1300 pM for the AA. In comparison to CESI, this work demonstrated up to 800‐fold sensitivity improvement. Its utility was showcased by analyzing an extract from a single HeLa cell, and a total of 450 peaks were observed, among which 20 AA were quantified and 40 metabolites identified (Fig. 4B).

The aforementioned technical advancements have rendered it possible to probe cationic metabolites in ultrasmall biological samples, even single cells, however, the application of CE‐MS for anionic metabolic profiling (i.e., acidic metabolites) still needs further improvement due to several issues. One of these issues is the frequent onset of corona discharge when coupling high‐pH separation conditions to negative ionization mode in nano‐ESI‐MS. Zhang et al. [30] proposed a “wrong way round” ionization approach of detecting nucleotides as [M+H]^+^ form by sheathless CE‐MS, thereby totally circumventing corona discharge. A mixture of 12 nucleotides was separated using 16 mM ammonium acetate (pH 9.7) as BGE in normal polarity, assisted by a forward pressure of 1 psi, and then detected in positive ionization mode. Hydrodynamic injection of a series of standard solutions revealed LOD values in the range of 0.06 to 1.33 nM, corresponding to 0.4 to 8.6 Amol of absolute amount injected. As for its biological application, intracellular nucleotides from HepG2 cells were quantified with the proposed method after sample preparation including ultrafiltration with 3 kDa cutoff membrane and liquid‐liquid extraction. The method validation of the proposed workflow demonstrated satisfactory results for linearity, precision, accuracy, and matrix effect. To demonstrate its usefulness in analyzing ultrasmall biological samples, cell pellets were diluted to a series of cell content densities for sequential sample preparation and analysis, with the lowest cell lysate corresponding to 500 cells only. Even in an extract of 500 HepG2 cells, the method enabled the detection of seven of the investigated nucleotides. This work also showed that the detection sensitivity could be further enhanced with transient isotachophoresis (tITP). However, the actual utility of the proposed method for metabolic profiling of intrinsically biomass‐limited samples still needs to be evaluated.

The vast structural differences and varying charge states of metabolites usually render it necessary to conduct multiple analytical runs, and it is desired to include diverse types of compounds in a single run. In order to boost the sensitivity and expand the metabolic coverage, Huang et al. [31] employed a multifunctional derivatization protocol for both organic acids and AA. The authors tested this derivatization strategy with dried analyte standards (21 compounds). Anhydrous pyridine and 3‐(Diethylamino) propinoyl chloride were first added for the first reaction, which was then quenched by adding *N, N*‐diethylethylenediamine, followed by the addition of Hexafluorophosphate Azabenzotriazole Tetramethyl Uronium (HATU) for the second reaction. Last, ammonium formate buffer was added for dilution prior to analysis by sheathless CE‐MS. The dual tagging strategy results in amide bond formation and ester formation that are both stable and resistant to hydrolysis. An in‐house fabricated sheathless CE‐MS interface was used for separation and 5 mM ammonium formate 10% methanol (pH = 2.5) selected as BGE, with MS operating in positive mode. The obtained LOD values for the metabolites investigated ranged from 9 to 225 nM. Not only did this one‐pot two‐step derivatization expand the detection of metabolites, but it also improved the overall detection sensitivity. The application of this proposed workflow was successfully showcased in a biological context with either human tissue or mammalian cells. For an injected fraction that corresponds to 267 cells, all of the investigated metabolites could be detected with an average *S*/*N* of 19. Authors proposed that their strategy could also be expanded to other metabolite classes.

CZE has been used as the main separation mode in CE‐MS‐based metabolomics studies, thereby primarily targeting hydrophilic/polar ionogenic metabolites. To expand the metabolic coverage space of CE‐MS, other separation modes may be considered. In this context, nonaqueous CE, in which BGEs are composed of organic solvents containing volatile salts, such as ammonium acetate in a small portion of water, has interesting features for the analysis of nonpolar and charged compounds. Moreover, the use of high organic solvent‐based BGEs may further improve the ESI efficiency. Recently, Azab et al. developed a high‐throughput nonaqueous capillary electrophoresis (NACE‐MS) method [16], employing multisegment injection for the profiling of more than 20 nonesterified FAs in human serum and plasma. This work utilized a BGE that consisted of 35 mM ammonium acetate in a mixture of ACN:methanol:water:isopropanol (70:15:10:5, v/v/v/v) with an apparent pH of 9.5 adjusted with adding of 12% ammonium hydroxide. Serum or plasma extracts were injected hydrodynamically at 50 mbar alternating between 5 s for each sample plug and 40 s for the BGE spacer plug for a total of seven distinct samples analysed within one run. Such a setup allows the QC samples to be measured within every run for technical variance assessment, and more importantly, for long‐term signal drift adjustment when analyzing large sample cohorts. An additional pressure of 20 mbar on the sample inlet was proven to be most beneficial when performing NACE, ensuring the resolution of very long‐chain FAs from the EOF, while maintaining good peak shapes and relatively short analysis time. The separated FAs were detected in negative ESI‐MS mode, where the authors employed a moderate sprayer voltage to avoid corona discharge. A simple extraction protocol using methyl‐tert‐butyl‐ether (MTBE) was adopted as the sample handling strategy, and this workflow was then subjected to rigorous method validation process, demonstrating comparable sensitivity to conventional GC methods. A major advantage of the proposed workflow enables rapid yet comprehensive profiling of FAs in volume‐limited biological samples, however, its capability of resolving certain positional and geometric isomers is still lacking.

## 3 Applications

The applicability of CE‐MS for metabolomics in various fields was demonstrated in 58 publications in the period from July 2018 to June 2020. The search terms “metabolomics,” “metabolic profiling,” “metabolic fingerprinting,” “capillary electrophoresis and mass spectrometry” were used for selecting these studies from ISI Web of Science and PubMed databases. An overview of these studies is given in Table 1, which provides information about the type of sample and compounds analyzed, the BGE, sample pretreatment procedure, the MS analyzer employed, LOD (when provided by the authors), and remarks on the type of approach, the type of capillary coating, and whether CE was used as a complementary method. In the following sections, some representative CE‐MS‐based metabolomics studies are discussed in more detail.

**Table 1:** Overview of CE‐MS‐based metabolomics studies reported between July 2018 and June 2020

Compounds	Sample matrix	BGE	Sample pretreatment	MS analyzer	LOD	Remarks	Ref.
Cationic metabolites	Human dried blood spot (DBS)	1 M formic acid, 15% ACN (pH 1.8)	Dried blood spot cut‐out specimen placed in 75% methanol and sonicated. Extraction solution filtered through 3kDa cutoff membrane. Evaporation and reconstitution.	QTOF	n.s.	Untargeted	[9]
Cationic and anionic metabolites	Human plasma and urine	1 M formic acid with 15% ACN (pH 1.8); 50 mM ammonium bicarbonate (pH 8.5)	Urine: dilution with water; Plasma: dilution with water, vortexed, and directly filtered through 3 kDa cutoff membrane	TOF & QTOF	n.s.	Targeted and Untargeted MSI CE‐MS; In combination with UPLC‐UV	[15]
Fatty acids	Human plasma and serum	35 mM ammonium acetate in 70% ACN, 15% methanol, 10% H_2_O, and 5% isopropanol (apparent pH of 9.5)	Extraction with MTBE/centrifugation/biphasic separation/	TOF	0.42 to 8.36 µM	Targeted and Untargeted MSI CE‐MS; In combination with CE‐UV	[16]
Cationic metabolites	Mouse plasma	10% acetic acid	LLE with methanol/water/ chloroform. Centrifugation and supernatant filtered through 3 kDa cutoff membrane. Evaporation and reconstitution.	TOF	n.s.	Untargeted	[24]
Cationic metabolites	HepG2 cells	10% acetic acid	LLE with methanol/water/chloroform. Supernatant filtered through 5 kDa cutoff membrane and evaporated	TripleTOF	1.4 to 9.2 nM (aspartic acid 417 nM)	Untargeted	[25]
Cationic metabolites	Mouse kidney tissue	10% acetic acid	Mixed with 75% methanol. Sonication/centrifugation/evaporation/reconstitution	QqTOF	n.s.	Untargeted; Sheathless porous tip interface. Ultrasmall final volume (2.5 µL). Modified sample vial.	[27]
Cationic metabolites	Single neuron from *A. Californica*	1% formic acid	Isolated neurons washed with water and placed in methanol, evaporated and resuspended in IPA:ACN(0.8:0.2,V:V). Centrifugation/supernatant evaporation/ reconstitution.	QTOF	n.s.	Untargeted; FASI CE‐MS analysis	[28]
Cationic metabolites	Single Hela cell	10% acetic acid	Single cell collected and released in methanol. Sonication, evaporation and reconstitution	Triple‐quad and QTOF	majority<20 pM. Glycine (100 pM) and aspartate (1300 pM).	Targeted and Untargeted; Large volume sample injection (circa 1200 nL) with dual preconcentration by isotachophoresis and stacking. In‐house CE‐MS interface design nanoCESI	[29]
Nucleotides	HepG2 cells	16 mM NH_4_Ac (pH 9.7)	Quenching with 80% methanol; cell lysate filtered by 3 kDa cutoff membrane. LLE with water/chloroform/methanol. Supernatant evaporated and reconstituted.	Triple TOF	0.1 to 0.9 nM	Targeted; Sheathless porous tip interface; Nucleotides analyzed in positive MS mode to circumvent corona discharge.	[30]
Cationic metabolites	Bovine aortic endothelial cells and human muscle	5 mM ammonium formate 10% methanol (pH = 2.5)	Cells: Quenching/sonication/ centrifugation/ supernatant evaporation/derivatization. Muscles: Homogenization/ centrifugation/supernatant evaporation/derivatization.	Quadrupole Ion Trap	9 to 187 nM	Targeted approach with derivatization; Home‐made sheathless CE‐MS interface.	[31]
Neurotransmitters	Rat brain stem/ whole *Drosophila*	50 mM ammonium formate (pH 2.5) in 40% methanol	Rat stem: Homogenization and centrifugation. Supernatant evaporated. Resuspension and dividing into aliquots. Mixed with ISTD Whole Fly: Homogenization centrifugation and supernatant divided to aliquots. Mixed with ISTD and evaporated. Reconstitution.	Triple Quad	10 pM	Targeted; Injection by electrokinetic supercharging; 5000‐fold improvement in detection limits compared to CZE with hydrodynamic injection	[32]
Cationic and anionic metabolites	Human plasma	10% acetic acid	LLE with methanol/water/ chloroform. Centrifugation and supernatant filtered through 3 kDa cutoff membrane. Evaporation and reconstitution.	TOF; TripleTOF	n.s.	Targeted	[33]
Cationic and anionic metabolites	Human brain tissue	1 M formic acid(pH 1.8); 50 mM ammonium acetate (pH 9.0)	Homogenization thrice in ACN/Water. Centrifugation, supernatant filtered through 5 kDa cutoff membrane and evaporation	TOF	n.s.	Targeted	[34]
Cationic metabolites	Human urine	0.5 M formic acid	10 fold dilution with water; filtered through 0.22 µM membrane	Triple Quad	0.22 to 8.73 µM	Targeted	[35]
Cationic and anionic metabolites	Human breast cancer biopsies	1 M formic acid(pH 1.8); 50 mM ammonium acetate (pH 8.5)	Homogenization and centrifugation. Supernatant filtered through 5 kDa cutoff membrane.	TOF	n.s.	Targeted	[36]
Cationic and anionic metabolites	Seaweed species	1 M formic acid; 50 mM ammonium acetate (pH 8.5)	LLE with methanol/water or methanol/water/chloroform. Centrifugation and supernatant filtered through 5 kDa cutoff membrane. Evaporation and reconstitution	TOF	n.s.	Targeted; In combination with LC‐MS/MS; Anionic metabolites separated on a cationic polymer coated SMILE (+) capillary	[37]
Cationic and anionic metabolites	Yeast	1 M formic acid; 50 mM ammonium acetate (pH = 9)	Extracted with boiling ethanol (75%). Vortexing/centrifugation/supernatant evaporation and reconstitution.	TOF	n.s.	Untargeted	[38]
Cationic and anionic metabolites	Mouse intestinal tracts	1 M formic acid; 50 mM ammonium acetate (pH = 8.5)	Intestinal contents run with PBS/mixed with methanol/vortexing/centrifugation/supernatant filtered through 5 kDa cutoff membrane/evaporation/reconstitution	TOF	n.s.	Untargeted; Anionic metabolites separated on Cosmo(+) capillary, chemically coated with a cationic polymer	[39]
Cationic and anionic metabolites	Beef muscles	1 M formic acid; 50 mM ammonium acetate (pH = 8.5)	Homogenization in ACN/water, centrifugation, supernatant filtered through 5 kDa cutoff membrane. Lyophilization and reconstitution.	TOF	n.s.	Targeted and Untargeted	[40]
Cationic metabolites	Vinegar	30 mM 18C6H4 in water	Filtered through 3 kDa cutoff membrane and diluted.	TOF	0.07 to 1.03 µg/mL	Targeted	[41]
Cationic and anionic metabolites	Human saliva	1 M formic acid; 50 mM ammonium acetate (pH 8.5)	Vortexed and filtered through 5 kDa cutoff membrane. Mixed with ISTDs for analysis	TOF	n.s.	Untargeted; Anionic metabolites separated on Cosmo(+) capillary, chemically coated with a cationic polymer	[42]
Cationic metabolites	Intracellular and extracellular fluids from HK‐2 cells	1 M formic acid pH 1.8	Intracellular: Methanol extraction, centrifugation, and supernatant evaporated Extracellular: Mixed with methanol, centrifugation, supernatant evaporated and reconstituted	QTOF	n.s.	Untargeted;	[43]
Cationic metabolites	Human plasma	10% acetic acid	Protein precipitation using TCA and then a parallel electromembrane extraction	TOF	n.s.	Targeted; Increasing the metabolite extraction throughput by parallel electromembrane extraction	[44]
Cationic metabolites	Bone marrow‐derived macrophages from mice	1 M formic acid in 10% methanol	Macrophages in methanol/H_2_O. 4*Frost/Defrosting cycles. Cells disruption. Centrifugation and supernatant evaporated and reconstituted.	TOF	n.s.	Untargeted;	[45]
Cationic and anionic metabolites	*Synechococcus* sp. PCC 7002	1 M formic acid (pH 1.8); 50 mM ammonium acetate (pH 9.0)	LLE with methanol/chloroform/ water. Supernatant filtered through 5 kDa cutoff membrane. Evaporation and reconstitution.	TOF	n.s.	Untargeted; In combination with GC‐MS	[46]
Cationic metabolites	Mouse liver	0.8 M formic acid in 10% methanol	Homogenization in water/methanol, centrifugation. Supernatant evaporation and reconstitution.	TOF	n.s.	Untargeted; In combination with GC‐MS and LC‐MS	[47]
Cationic and anionic metabolites	Human plasma	1 M formic acid; 50 mM ammonium acetate (pH 9.0)	LLE with methanol/chloroform/ water. Supernatant filtered through 5 kDa cutoff membrane. Evaporation and reconstitution.	TOF	n.s.	Targeted	[48]
Cationic metabolites	Human plasma	10% acetic acid	LLE with methanol/water/ chloroform. Supernatant filtered through 5 kDa cutoff membrane and evaporated.	TOF	n.s.	Untargeted	[49]
Cationic and anionic metabolites	Prostate cell lines	1 M formic acid (pH 1.8); 50 mM ammonium acetate (pH 8.5)	Quenching with liquid nitrogen. LLE with methanol/water/chloroform. Supernatant filtered through 5 kDa cutoff membrane and evaporated	TOF	n.s.	Targeted and untargeted	[50]
Cationic metabolites	Cell culture media	0.8 M FA in 10% methanol	Mixed with ACN containing formic acid. Vortexed and filtered through 30 kDa cutoff membrane.	TOF	n.s.	Untargeted; In combination with LC‐MS	[51]
Cationic and anionic metabolites	MEF cells and Human HCC cell lines	1 M formic acid (pH 1.8); 50 mM ammonium acetate (pH 8.5)	LLE with methanol/water/chloroform. Centrifugation. Supernatant filtered through 5 kDa cutoff filter and evaporated	TOF	n.s.	Targeted; In combination with GC‐MS; The fused capillary first preconditioned with an phosphate‐containing electrolyte to mask the dissociated silanol groups inside the capillary prior to anionic profiling	[52]
Cationic and anionic metabolites	Human OSCC cell line	1 M formic acid; 50 mM ammonium acetate (pH = 8.5)	Quenching with methanol. LLE with methanol/water/chloroform. Supernatant filtered through 5 kDa cutoff membrane and evaporated.	TOF	n.s.	Untargeted; anionic metabolites separated on Cosmo(+) capillary, chemically coated with a cationic polymer	[53]
Cationic metabolites	Peritoneal macrophages/cells	1.0 M formic acid in 10% methanol	Double extraction with 3 freeze/thaw cycles. Sonication, vortexing, and centrifugation. Supernatant evaporated	TOF	n.s.	Untargeted; In combination with GC‐MS and LC‐MS	[54]
Cationic and anionic metabolites	Human serum	1 M formic acid; 50 mM ammonium acetate (pH = 8.5)	LLE with methanol/water/chloroform. Supernatant filtered through 5 kDa cutoff membrane. Evaporation and reconstitution.	TOF	n.s	Untargeted; anionic metabolites separated on Cosmo(+) capillary, chemically coated with a cationic polymer	[55, 56]
Cationic and anionic metabolites	Mice fecal samples	1 M formic acid; 50 mM ammonium acetate (pH = 8.5)	Samples in methanol/water. Homogenization/centrifugation/ultrafiltration 5 kDa cutoff membrane/evaporatio/reconstitution	TOF	n.s.	Untargeted; anionic metabolites separated on Cosmo(+) capillary, chemically coated with a cationic polymer	[57]
Cationic and anionic metabolites	Brown adipose tissue from rats	1 M formic acid; 50 mM ammonium acetate (pH = 8.5)	Mixed in ACN and water/homogenization/centrifugation/ supernatant filtered through 5 kDa cutoff membrane/evaporation/reconstitution	TOF	n.s.	Untargeted; In combination with LC‐MS	[58]
Cationic and anionic metabolites	Human plasma	1 M formic acid; 50 mM ammonium acetate (pH = 8.5)	LLE with methanol/water/ chloroform and supernatant filtered through 5 kDa cutoff membrane. Evaporation and reconstitution.	TOF	n.s.	Untargeted; In combination with LC‐MS; anionic metabolites separated on Cosmo(+) capillary, chemically coated with a cationic polymer;	[59]
Cationic and anionic metabolites	C2C12 mouse myoblasts	1 M formic acid; 50 mM ammonium acetate (pH = 7.5)	LLE with methanol/water/ chloroform and supernatant filtered by 5 kDa cutoff membrane. Evaporation and reconstitution.	TOF	n.s.	Untargeted; anionic metabolites separated on a GC capillary, poly(dimethylsiloxane) (DB‐1)	[60]
Cationic and anionic metabolites	Uterine serous carcinoma (USC) cells	1 M formic acid; 50 mM ammonium acetate (pH = 8.5)	Extraction with methanol/water. Centrifugation/supernatant filtered through 5 kDa cutoff membrane/evaporation and reconstitution.	TOF	n.s.	Targeted; anionic metabolites separated on Cosmo(+) capillary, chemically coated with a cationic polymer	[61]
Cationic and anionic metabolites	Human tumor tissues and adjacent normal tissues	1 M formic acid; 50 mM ammonium acetate (pH = 8.5)	Mixed in ACN and water/homogenization/centrifugation/supernatant filtered through 5 kDa cutoff membrane/evaporation/reconstitution	TOF	n.s.	Untargeted; anionic metabolites separated on Cosmo(+) capillary, chemically coated with a cationic polymer	[62]
Cationic and anionic metabolites	Rat kidney	1 M formic acid; 50 mM ammonium acetate (pH = 8.5)	Homogenization. Mixing with 70% cold ACN/vortexing/centrifugation	TOF	n.s.	Targeted and untargeted; In combination with HPLC	[63]
Cationic and anionic metabolites	Human serum	1 M formic acid; 50 mM ammonium acetate (pH = 8.5)	LLE with methanol/water/chloroform. Centrifugation and supernatant filtered through 5kDa cutoff membrane. Evaporation and reconstitution.	TOF	n.s.	Untargeted	[64]
Cationic and anionic metabolites	Dolphin and beagle dog plasma	1 M formic acid; 50 mM ammonium acetate (pH = 8.5)	LLE with methanol/water/chloroform. Supernatant filtered through 5 kDa cutoff membrane. Evaporation and reconstitution.	TOF	n.s.	Untargeted; In combination With LC‐MS	[65]
Cationic and anionic metabolites	Mouse plasma	1 M formic acid; 50 mM ammonium acetate (pH = 8.5)	LLE with methanol/water/chloroform. Supernatant filtered through 5kDa cutoff membrane. Evaporation and reconstitution.	TOF	n.s.	Targeted; anionic metabolites separated on a cationic polymer coated SMILE (+) capillary	[66]
Cationic and anionic metabolites	Mice fecal samples	1 M formic acid; 50 mM ammonium acetate (pH = 8.5)	Diluted in PBS and extracted thrice by vortexing/resting. Supernatant centrifuged and filtered through 5 kDa cutoff membrane	TOF	n.s.	Untargeted; anionic metabolites separated on a cationic polymer coated SMILE (+) capillary	[67]
Cationic and anionic metabolites	Caco‐2 cells	1 M formic acid; 50 mM ammonium acetate (pH = 8.5)	Quenching and LLE with methanol/water/chloroform. Supernatant filtered through 5 kDa cutoff membrane. Evaporation and reconstitution.	TOF	n.s.	Targeted;	[68]
Cationic and anionic metabolites	Mouse skeletal muscle	1 M formic acid; 50 mM ammonium acetate (pH = 8.5)	LLE with methanol/water/chloroform. Supernatant filtered through 5kDa cutoff membrane. Evaporation and reconstitution.	TOF	n.s.	Untargeted;	[69]
Cationic and anionic metabolites	Human skeleton muscle	1 M formic acid with 15% ACN (pH 1.80); 50 mM ammonium bicarbonate (pH 8.50)	Two‐step LLE with methanol/water/chloroform. Two batches of supernatant combined.	TOF	n.s.	Untargeted; In combination with CE‐UV	[70]
Cationic metabolites	Mouse lung	1 M formic acid in 10% methanol	Homogenized and mixed with formic acid. Centrifugation and supernatant filter sterilized through 0.22 uM spin‐x columns. Evaporation and reconstitution.	TOF	n.s.	Untargeted; In combination with LC‐MS and GC‐MS	[71]
Cationic metabolites	Mouse serum	1 M formic acid	LLE with methanol/water/ chloroform. Centrifugation and supernatant filtered through 5kDa cutoff membrane. Evaporation and reconstitution.	QTOF	n.s.	Untargeted and targeted;	[72]
Cationic metabolites	Human plasma	1 M formic acid in 10% methanol	Mixed with 0.2 M formic acid (5% ACN), and filtered through 30 kDa cutoff membrane.	TOF	n.s.	Untargeted; In combination with LC‐MS and GC‐MS	[73]
Cationic metabolites	Rat brain microdialysis sample	10% acetic acid	Directly diluted (1:1,v/v) with BGE	TOF	6.2 to 70 nM in water; 11 to 284 nM in perfusate	Untargeted and targeted	[74]
Cationic and anionic metabolites	Rice plants	1 M formic acid; 50 mM ammonium acetate (pH 6.9)	LLE with methanol/water/ chloroform. Centrifugation and supernatant combined.	TOF	n.s.	Untargeted; In combination with GC‐MS; Anionic metabolites separated on a cationic polymer coated SMILE (+) capillary	[75]
Cationic and anionic metabolites	Tobacco plants	1 M formic acid; 50 mM ammonium acetate (pH 8.5)	LLE with methanol/water/chloroform. Centrifugation and supernatant filtered through 5 kDa cutoff membrane. Evaporation and reconstitution	TOF	n.s.	Untargeted; In combination with LC‐MS and GC‐MS	[76]
Cationic and anionic metabolites	HK‐2 cell	1 M formic acid; 50 mM ammonium acetate (pH 8.5)	LLE with methanol/water/chloroform. Centrifugation and supernatant filtered through 5 kDa cutoff membrane. Evaporation and reconstitution	TOF & Triple Quad	n.s.	Targeted	[77]
Polar/ionic metabolites; Fatty acids and bile acids	Mouse placental specimens	1 M formic acid with 13% ACN (pH 1.8); 50 mM ammonium bicarbonate (pH 8.5); 35 mM ammonium acetate (pH 9.5) in 70% ACN, 15% MeOH, 5% IPA, and 10% water	Polar/ionic metabolites: Freeze‐drying followed by LLE with methanol/water/ chloroform. Mix with internal standards prior to analysis. Fatty acids and bile acids: Hydrolysis of lipids followed by MTBE extraction. Supernatant evaporation and reconstitution	TOF	n.s.	Untargeted and targeted; MSI CE‐MS	[78]
Polar/ionic metabolites; Fatty acids and bile acids	Human serum	1 M formic acid with 13% ACN (pH 1.8); 35 mM ammonium acetate (pH 9.5); 35 mM ammonium acetate (pH 9.5) in 70% ACN, 15% MeOH, 5% IPA and 10% water	Polar/ionic metabolites: Freeze‐drying followed by LLE with methanol/water/chloroform. Mix with internal standards prior to analysis. Fatty acids and bile acids: Hydrolysis of lipids followed by MTBE extraction. Supernatant evaporation and reconstitution	TOF	n.s.	Untargeted; MSI CE‐MS	[79]

n.s., not stated.

### 3.1 Biomedical and clinical applications

Wells et al. developed a MS‐compatible electrokinetic supercharging (EKS) strategy for the sensitive and robust analysis of biogenic amines in biological samples [32]. This work utilized a low pH BGE that consists of 50 mM ammonium formate (pH 2.5) and 40% methanol, and a leading electrolyte (LE) that contained 250 mM ammonium formate (pH 2.5). A conventional sheath‐liquid interface was used for coupling CE to MS. To perform EKS, hydrodynamic injection of LE at 50 mbar for 30 s and water at 50 mbar for 1 s was first conducted, followed by electrokinetic injection of sample at 30 kV for 150 s. LOD values down to 10 pM were obtained for the investigated neurotransmitters in EKS, resulting in a 5000‐fold sensitivity enhancement compared to hydrodynamic injection. The utility of this method was demonstrated in the determination of several neurotransmitters in rat brain homogenate and whole Drosophila homogenate, emphasizing its capability of simultaneously measuring pM and µM concentrations. Furthermore, the authors compared quantified concentrations of the amines in rat brain homogenate by the EKS method with those by an existing LC‐MS/MS method. Greater differences (20–46%) between the two analytical approaches were discovered in concentration for compounds containing moieties prone to oxidation. It was speculated that more rapid analysis and efforts in oxidation prevention may help to further increase the accuracy of the proposed CE‐MS method.

The field of nanomaterial (NM) corona is predominately focused on the adsorption of proteins to the NM surface, and has only seen a few studies investigating a subset of the metabolome. Insights into the interactions between metabolites and NMs can help form a comprehensive understanding of the role of the corona in determining the biological consequences of NMs. To study metabolite recruitment characteristics on the surface of NMs, Chetwynd et al. employed CE‐MS with both sheathless and sheath‐liquid interfaces [33]. Low‐pH separation conditions were used throughout the whole study, with normal CE polarity for cationic detection and reverse CE polarity for anionic detection. Targeted compounds were incubated together with six biologically relevant NMs in water, and the effect of proteins on the adsorption characteristics was also investigated by introducing either intact or protein‐free plasma in the incubation mixture. Since it is difficult to directly analyze the depleted amount of metabolites, the authors analyzed the remainder fraction in the supernatant after incubation. Unique adsorption characteristics were demonstrated for different NMs while the mechanism underlying such interactions was still unclear. Metabolite recovery studies were conducted by using three rounds of washing steps, however, although the recovery data clearly indicated the amount of metabolites not detected in the supernatant was adsorbed to the NMs, the adsorbed amount could not be fully recovered. By carrying out incubation experiments with either intact or protein‐free plasma, the authors illustrated that protein portion of the corona is essential to the formation of the metabolite corona to form a complete biomolecular corona. Despite the discoveries of many intriguing phenomena, this pilot study generated plenty of unanswered questions that require more follow‐up research.

The pathogenesis of Alzheimer's disease (AD) has been shown to involve dysregulation in multiple biochemical pathways. To test whether dysregulation of choline‐related biochemical pathways in the brain are related to AD pathogenesis, Mahajan et al. performed targeted and quantitative CE‐MS metabolomics analysis on human brain tissue samples and transcriptomics study [34]. CE‐MS analyses were conducted according to the protocol from Human Metabolome Technologies (HMT) for both cations and anions. This work focused on 26 quantified metabolites that represented biochemical reactions associated with transmethylation and polyamine synthesis/catabolism mainly in two brain regions, inferior temporal gyrus (ITG) and middle frontal gyrus (MFG). Significant alterations in metabolites (mainly in the inferior temporal gyrus [ITG]), such as choline, S‐adenosyl methionine, cysteine, reduced glutathione (GSH), spermidine, *N*‐acetyl glutamate, *N*‐acetyl aspartate, and gamma‐Aminobutyric acid (GABA) distinguished AD from control groups, and were also indicative of severity of AD pathology. However, the transcriptomic analyses focused on other brain regions, which hindered its integration with metabolomics data. Additionally, the findings in this work need to be further validated in studies with larger sample sizes.

**Figure 5:**
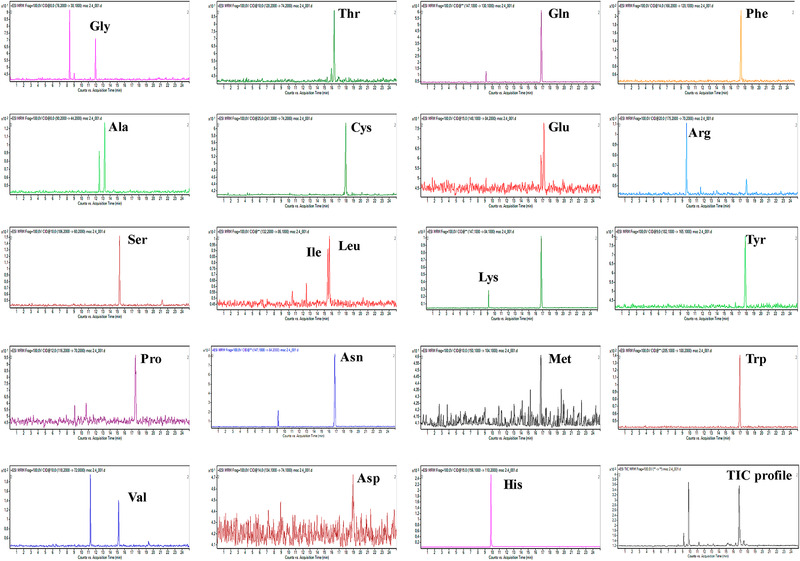
Extracted analytical profiles of clinical sample. Multiple reaction monitoring (MRM) transitions from the CE‐MS/MS analysis of a 10‐fold diluted human urine sample obtained from a patient suffering from inflammatory bowel disease undergoing treatment with thiopurines (azathioprine). Reproduced from Ref. [35] with permission.

Recently, Piestansky et al. developed a CE‐MS/MS method for the analysis of AA in urine samples of inflammatory bowel disease (IBD) patients [35]. A sheath‐liquid interface was used for coupling CE to a Triple Quadrupole tandem MS instrument. A 500 mM formic acid solution was selected as BGE for the separation of 20 proteinogenic AA. The method evaluation revealed satisfactory performance parameters, and yielded LOD values of 0.22 to 8.73 µM in urine. The validated CE‐MS/MS method was then applied for the targeted analysis of AA in urine from Crohn's disease patients who were treated with azathioprine, and representative extracted profiles are shown in Fig. 5. The obtained concentrations of AA in urine were normalized to that of creatinine, and showed high consistency with the data produced by a UHPLC‐MS system. This emphasized the potential of CE‐MS as an economic and reliable alternative to UHPLC‐MS for clinical routine AA monitoring. The statistical analysis of the data revealed a moderate decrease in seven AA in IBD patients when compared to the healthy control subjects.

**Figure 6:**
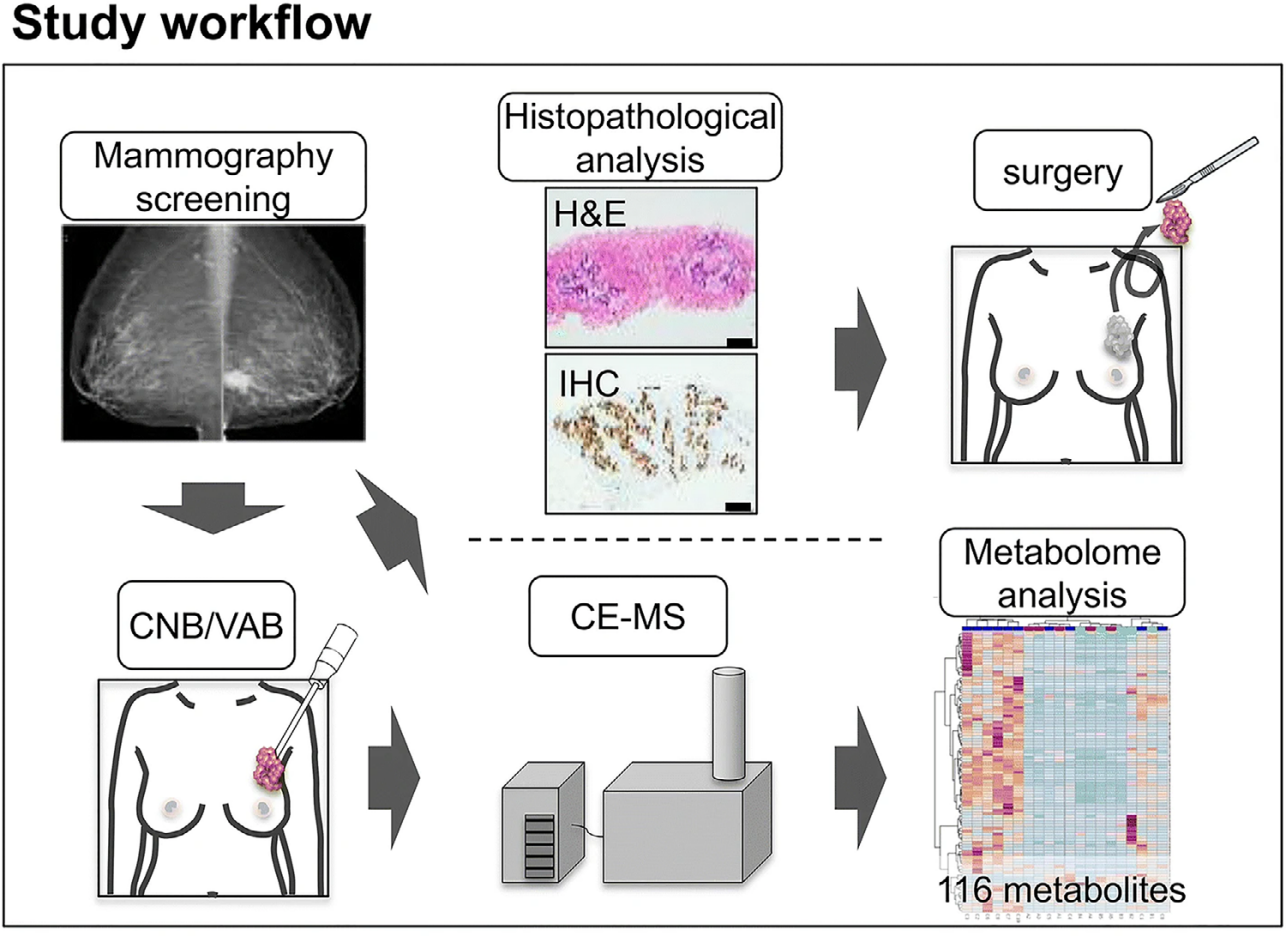
A schematic overview of the workflow of the study. Reproduced from Ref. [36] with permission.

Precision medicine is an approach in disease treatment that enables accurate therapy and avoids drug resistance, and one such example is the routinely practiced subtype‐based molecular targeted therapy for breast cancer patients. However, the existing breast cancer stratification approaches lack the accuracy in predicting drug resistance and prognosis. To improve breast tumor stratification strategies, Harada‑Shoji et al. employed CE‐MS‐based metabolomics profiling using breast biopsy samples [36]. An average of 25 mg biopsy sample was obtained from patients with benign tumors, ductal carcinoma *in situ* (DCIS), or invasive ductal carcinoma (IDC) (Fig. 6). The CE‐MS profiling of 116 metabolites engaged in central metabolism in the collected biopsy samples eventually resulted in a collection of 97 quantified metabolites for further data processing. Statistical analysis unveiled a unique metabolic signature of “pure” IDC cases, while that of DCIS showed similarity to benign samples. When compared to the rest, the “pure” IDC cases presented significantly increased levels of 89 metabolite concentrations. Pathway analysis revealed pyrimidine metabolism to be the most affected one in “pure” IDC samples. This study emphasized the feasibility of CE‐MS‐based metabolomics in a clinical setting and how it may help improve breast cancer stratification. Meanwhile, further exploration is still required to explain the heterogeneity in metabolic profiles within groups, and to link metabolic information with prognosis in the clinical setting.

### 3.2 Plant and microbial applications

To comprehensively characterize water‐soluble metabolites in three major seaweed groups and explore the influence of different extraction protocols on metabolic profiles, Hamid et al. utilized a cross‐platform metabolomics approach with both LC‐MS/MS and CE‐MS [37]. This study included three groups, 11 algae species. Prior to instrumental analyses, authors conducted two different metabolite extraction protocols using methanol‐water, with and without chloroform. CE‐MS analysis was employed for both cationic and anionic profiling, focusing on free AA, organic acid, and charged metabolites, while LC‐MS/MS approach was adopted for the analysis of free sugars. It was revealed that most AA could be found in all the tested species, with their concentration differences mainly attributed to their algae groups. Among the AA, alanine was found to be the most abundant in all algae species, which is a reflection of the metabolism change caused by the oxygen flux as a consequence of high and low tide in the sea. The obtained metabolite profiles from LC‐MS and CE‐MS were subjected to multivariate analysis, revealing clear inter‐ and intragroup distinctions in the principal component analysis (PCA) plots. However, clustering analysis showed that no seaweed group characteristics with AA were uncovered. Instead, the sugar profiles demonstrated a characteristic alignment with the taxonomy tree representing three seaweed group. Although some effect of the extraction method was observed on the compound concentrations, the extent of such effect was very limited on the outcome and the differences in the metabolite profiles obtained were mainly the result of interspecies dissimilarities.

It was reported that *Scheffersomyces stipitis* uses *N*‐acetyl‐d‐glucosamine (GlcNAc) as its sole carbon source, but the GlcNAc metabolic pathway in *S. stipitis* is still poorly understood. In order to investigate the metabolic responses to GlcNAc in *S. stipitis*, Inokuma et al. conducted both cationic and anionic analyses on a CE‐MS system with sheath‐liquid interface [38]. The harvested yeast cells were first subjected to anaerobic fermentation for 24 h prior to metabolite extraction using a boiling ethanol method at 95°C for 5 min. This study verified over 130 metabolites in total, among which were 106 compounds associated with carbon and nitrogen metabolism. The PCA demonstrated clear separation between the metabolic data of yeast cells cultivated with GlcNAc and those with xylose and glucose. The GSSG/GSH ratio, an indicator of intracellular oxidative stress, was found to be approximately twofold higher in GlcNAc‐grown cells than in glucose‐grown cells, indicating the former are exposed to high levels of oxidative stress. A wide range of nitrogen‐containing compounds, such as AA, purines, and pyrimidines, showed increased accumulation during GlcNAc assimilation in *S. stipitis*. The qRT‐PCR showed that the increase of AA in GlcNAc‐grown cells was due to the induction of expression of five genes responsible for encoding certain AA synthases, while the elevated concentrations of purine and pyrimidine could be attributed to the utilization of ammonia as the amino donor for glutamine‐dependent amidotransferases that help the biosynthesis of various purines and pyrimidine intermediates. However, future work is required to characterize these amidotransferases in *S. stipitis*. Interestingly, many of these nitrogen‐containing compounds are valuable due to their pharmaceutical properties, which potentially could render *S. stipitis* a tool for the direct production of these compounds from GlcNAc.

The different composition of gastrointestinal microbiota across the intestinal tract reportedly contributed to the difference between the small and large intestinal metabolome profiles, however, little is known about the metabolome profiling throughout the gastrointestinal tract and its correlation with gastrointestinal microbiota. To gain more insight into this, Yamamoto et al. conducted metabolomics assays using both CE‐MS and LC‐MS/MS for the measurement of gastrointestinal luminal metabolite concentrations across different sections of the intestinal tract in specific pathogen‐free (SPF) and germ‐free (GF) mice [39]. CE‐MS‐based metabolic profiling included both cationic and anionic metabolites. Cationic metabolites were separated on fuse‐silica capillaries in low‐pH condition while the separation of anionic metabolites was done on a COSMO(+) capillary at high‐pH separation conditions. Metabolome analysis by CE‐MS and LC‐MS/MS identified a total of 382 metabolites in gastrointestinal luminal contents from SPF and GF mice. The results revealed a significantly higher number of gastrointestinal luminal metabolites in SPF mice than in GF mice. Significantly more metabolites from the upper and lower colon could be detected in SPF mice than in GF mice, suggesting that colonic microbiota may produce unique type of metabolites only in the former. The examination of the metabolome data obtained for different parts of the gastrointestinal tract in SPF and GF mice indicated that gut microbiota is responsible for the production of specific metabolites, and the signature of different metabolites between these two types of mice. Further exploration is still needed to better comprehend the contribution of gut microbiota to the gastrointestinal luminal metabolome.

### 3.3 Food applications

In meat industry, a comprehensive understanding of the alterations that take place in postmortem muscle metabolites could offer essential information on how to manipulate the production of key compounds to improve the quality of meat. Recently, Muroya et al. employed a sheath‐liquid CE‐MS approach in an attempt to find metabolites and pathways relevant to postmortem aging and beef quality in Japanese Black (JB) cattle [40]. Lean muscle pieces at the same position were taken from three steers at 0, 1 day, and 14 days postmortem and stored at −80°C until use. The frozen muscle pieces were homogenized in 50% 0°C ACN, followed by centrifugation and ultrafiltration through a 5 kDa cutoff membrane. The filtrate was subsequently lyophilized and reconstituted for both cationic and anionic profiling with HMT protocol. The CE‐MS‐based metabolomics study detected 197 compounds, of which 171 were annotated and 70 quantitated. Among the annotated metabolites, a total of 89 metabolites showed significant changes during postmortem aging of beef (adjusted *P* < 0.05, false discovery rate < 0.10). These metabolites were assigned to functional pathways and six of them were highlighted as the characteristic biochemical events related to meat quality including glycolysis, the citric acid cycle, the pentose phosphate pathway, protein digestion, amino acid generation, and purine metabolism. These progresses are expected to contribute to the quality improvement of aged beef, in aspects of tenderness, flavor, and functional value. Although significant changes in metabolite contents illustrated in this study agreed with previous studies of postmortem meat aging, the authors failed to consider the impact on postmortem metabolite generation by the variation of body weight among the tested animals.

**Figure 7:**
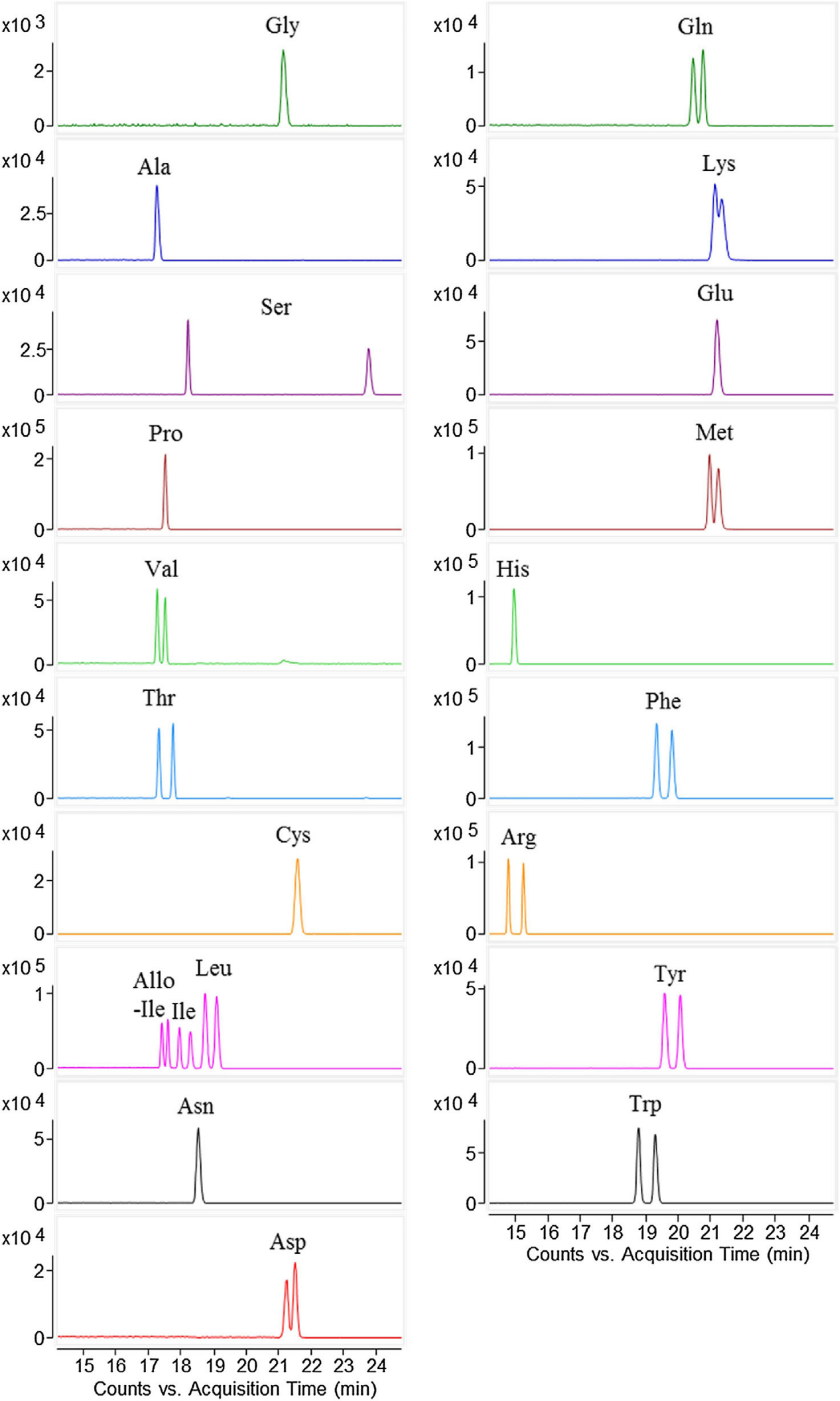
Extracted ion electropherograms of 20 d‐ and l‐amino acids (20 µg/mL) obtained with CE‐MS using 30 mM (+)‐18C6H4 as chiral selector and in water and employing partial filling injection. The separation buffer was injected at 50 mbar for 17 min to fill 70% of the capillary. Reproduced from Ref. [41] with permission.

To determine d‐AA in vinegars, Lee et al. developed a CE‐MS method for the enantiomeric separation of underivatized free AAs [41]. This work made use of a sheath‐liquid interface for the coupling of CE to MS and a separation buffer that consisted of 30 mM (18‐crown‐6)‐2,3,11,12‐tretracarboxylic acid (18C6H4), used as a chiral selector (CS). To minimize ESI contamination by the nonvolatile CS and improve the ionization efficiency, a partial filling approach was employed by filling approximately 70% of the capillary with the separation buffer after flushing with 1 M FA before hydrodynamic injection of the sample solution. In practice, AA enantiomers interacted with the CS and got separated before entering the FA segment and arriving to the ESI source. The separated AA peaks observed in this work were free AA ions, [AA+H]^+^ (Fig. 7). This approach provided resolution values (*Rs*) between 0.6 and 32.4 and LOD values from 0.07 to 1.03 µg/mL, suitable for detecting traces of D‐AAs in food. This method was then applied in the analysis of AA enantiomers in three types of vinegars, where little matrix effect was observed. D‐AAs could be detected in all the vinegars, taking up 0.4 to 2.0% of the total AAs determined. The varying abundance of D‐AAs in vinegars can indicate the extent of fermentation. Moreover, the method can potentially be utilized for assessing the taste profile of fermented foods based on the content of D‐AAs.

## 4 Conclusions and perspectives

Over the past 2 years, the applicability of CE‐MS for metabolomics studies in various fields was demonstrated in 58 publications, of which major fractions were focused on the global profiling of metabolites (Table 1). The CE‐MS‐based metabolomics studies revealed important findings in the various application areas as outlined in this article. Though the majority of the reported studies have been performed with CE‐MS methods employing a sheath‐liquid interface, there is a growing interest to use low‐flow or sheathless interfaces, especially with respect to further improving the detection sensitivity of CE‐MS for metabolomics. Especially, for biological questions dealing with severely limited sample amounts such as single (mammalian) cells. However, careful assessment of preanalytical steps, especially for checking loss of metabolites as a result of adsorption effects and aspects related to volume mismatch, that is, the sample is often further diluted during the preanalytical steps, is needed in this context. In the latter case, injection strategies need to be considered which allow to get the relevant fraction of the biomass‐restricted sample efficiently into the CE system. We anticipate that the use of preanalytics fully adapted to volume‐restricted samples with recently developed CE‐MS approaches will enable the reliable profiling of metabolites in low numbers of (mammalian) cells. These technological developments will be of high value to single cell biopsies and nanodosing studies. Overall, the studies reported here clearly show the unique capabilities of CE‐MS for (volume‐restricted) metabolomics studies.

*Drs. Rawi Ramautar and Wei Zhang would like to acknowledge the financial support of the Vidi grant scheme of the Netherlands Organization of Scientific Research (NWO Vidi 723.016.003)*.

*The authors have declared no conflict of interest*.
